# Online Pediatric Information Seeking Among Mothers of Young Children: Results From a Qualitative Study Using Focus Groups

**DOI:** 10.2196/jmir.6.1.e7

**Published:** 2004-03-01

**Authors:** Jay M Bernhardt, Elizabeth M Felter

**Affiliations:** ^1^Department of Behavioral Sciences and Health EducationRollins School of Public HealthEmory UniversityUSA; ^2^At the time of this research: Department of Health Promotion and BehaviorUniversity of Georgia, USACurrently: National Center for Chronic Disease Prevention and Health PromotionCenters for Disease Control and PreventionUSA

**Keywords:** Internet, Pediatrics, Focus Groups, Mothers, Health Education

## Abstract

**Background:**

Pre-natal and post-natal periods are times when many women actively seek health information from multiple sources, including the Internet. However, little is known about how pregnant women and mothers of young children seek and process online pediatric health information.

**Objective:**

To explore why and where mothers of young children look for online health information and how they determine if the information they receive is trustworthy.

**Methods:**

Focus groups were conducted in a Southeastern US city to provide an in-depth exploration of web-related behaviors and beliefs among mothers who work inside and outside of the home. Data from the focus groups were coded using deductive and inductive coding schemes and content was analyzed for the existence of themes.

**Results:**

Twenty mothers of young children participated in four focus groups. Most participants sought information on the Internet during pregnancy and nearly all sought online information after their child was born, primarily to diagnose or treat pediatric conditions and to seek advice on parenting and development. Participants mainly used commercial information websites for health information and many expressed disdain for commercial product websites. Many also expressed concerns about the reliability of health information on the web and described strategies for determining how much they trust each website.

**Conclusions:**

Women appear to be high information seekers during pregnancy and the first few years following delivery, and this period represents an important window of time for providing online health information. Participants suggested that online information sources and motives for providing online information should be clear in order to increase perceptions of trust. Participants expressed preference for online clinical health information that is presented by clinical professionals, and online parenting advice that is presented from other parents.

## Introduction

Pregnant women and mothers of young children are active consumers of health information about themselves and their children, and there are countless books, magazines, videos, television programs, classes, and other resources on childbirth, parenting, and pediatrics from which mothers and mothers-to-be can choose. An additional mass communication channel, the World Wide Web, has grown into a popular destination for women seeking health information on a wide range of pediatric topics.

The proliferation of websites offering pediatric health information is consistent with the growth of the broader universe of health information now available on the Internet. There are thought to be hundreds of thousands of health-related websites [1] and health information is one of the most researched topics among Internet users, [2] particularly among parents [3]. However, the high quantity of health related websites should not be confused with quality. Many studies have found that misleading and even patently false health information is rampant online [4-6]. Previous research with Internet users has found that most health-related searches start in general search engines and that numerous criteria, including many non-scientific judgments, are used by information seekers to assess the credibility of health-related websites [7]. However, little is known about Internet use among mothers of young children, how they use the web to retrieve pediatric health information, and how they make decisions about the veracity of the health information they receive.

The objective of this study was to explore web use preferences and perceptions among mothers of young children. Specifically, *why* they turn to the web for pediatric health information, *where* they go on the web for pediatric health information, and *how* they determine if the pediatric health information they receive online is trustworthy.

## Methods

### Design and Sample

This study was conducted in early 2001, in a medium-size metropolitan area in the Southeastern US with women recruited from three locations. Two of the locations were day-care facilities: one was affiliated with a governmental office and one was a private day-care facility. Both of these facilities provided full-time daycare and the mothers who were recruited through them held jobs for pay outside of the home. The third location from which women were recruited was a local "mother's center" frequented by mothers who mostly did not hold jobs outside of the home. Eligibility criteria included being at least 18 years of age, having at least one child younger than five, and using the Internet at least once in the previous 6 months.

Four focus groups were conducted: two groups with mothers who worked for pay outside the home and two groups with mothers who did not work outside the home at all, or who did so only part time. The focus groups were separated by participant work status so that mothers in each group would more freely share their experiences and perspectives. In addition, work status may affect people's Internet access and time available for going online. Although seven or eight women were recruited for each group, exactly five women participated in each. Additional focus groups were not conducted after convergence was found in the data across the four groups around the central research questions. Each focus group lasted about 90 minutes and study participants each received a $15 honorarium and complimentary childcare for their children during the focus groups. The study was approved by the Institutional Review Board at the host university and all participants provided written informed consent prior to participating.

### Measures

The focus groups were facilitated using a moderator guide that was developed using the "funnel approach" by beginning each discussion with less structured interview questions and moving to more focused questions and probes as the discussion continued [8]. The moderator guide was a female in her mid-20's, with extensive maternal and child health experience. The facilitator used standard focus group moderator guidelines [9] , and the topics that were covered included patterns of Internet use, processes for searching, preferences for types of pediatric health information on the Internet, and perceptions of the trustworthiness of health information sources. In addition to the open-ended questions and follow-up probes in the moderator guide, examples of websites that provide health information about pediatric sun protection were projected onto a screen and questions were asked about the participants' impressions and reactions to these sites. Please refer to the supplemental "Focus Group Guide" available online.

### Analysis

All data were collected prior to analysis. The focus group discussions were audiotaped, videotaped and transcribed verbatim, and a research assistant took detailed field notes during each discussion. The videotapes were used to assist with the assignment of specific participant speakers to the transcribed data. After each focus group, the research assistant and the moderator reviewed the field notes and came to consensus on the themes, issues, and ideas presented in the session. The coding scheme then was developed both inductively using a "cut and paste" technique with each research question [9] and deductively using relevant constructs from McGuire's Input/Output Matrix [10] as codes. The McGuire Matrix demonstrates the relationship between message factors or "inputs" (e.g., source, channel, receiver) and the resulting steps of receiving and processing messages or "outputs" (e.g., attention, liking, attitude change). The Ethnograph, [11] a qualitative data analysis program, was used for coding the data and facilitating analysis by generating code-specific reports from the data which were content analyzed for the existence of patterns and themes.

## Results

### Participants

A total of 20 women, ranging in age from 22 - 42 years, participated in the focus groups. The mean age was 34.5 (S.D.=4.95). Seventeen of the participants were Caucasian, two were African American, and one was Asian American. In addition, half of the participants had one child, eight participants had two children, and two participants had three or more children. Eighteen (90%) of the participants reported having Internet access at home and 80% of the mothers from the groups that worked outside the home reported having Internet access at work.

### Reasons for seeking pediatric health information

Almost all participants reported that during their pregnancy they had sought out information on the Internet about many pregnancy-related topics, and this was especially true for first pregnancies. Of particular interest to the mothers were websites that presented information on fetal development with content that was individually tailored to their due date or stage of pregnancy. Many participants also reported that they sought social support on the web from other pregnant women or mothers, especially during their first pregnancy. For example, one woman stated that "...I was so scared and I would go to this web site every day and just look at it (for) like ten minutes straight and I just found so much helpful information on it (about) being scared and not knowing what to expect." Several participants also shared the fact that they used the Internet to research specific problems they were having with their pregnancy.

The most common reasons that participants reported going online for health information following childbirth included: (1) wanting to learn more about diagnosing and treating specific pediatric health conditions, and (2) seeking out advice and support on parenting issues and development. Nearly every participant reported that she had used the Internet at least once to look up an illness or health condition that her child was experiencing. In many of these cases, the mothers were looking for information to clarify or supplement what they had been told by their doctors and to help them make more informed treatment decisions. For example, one mother whose son had chronic ear infections noted: "...I found so much information about how these things happen, which wasn't explained to me by his doctor. I found that very informative and felt like I knew as much or more than his pediatrician on the subject... And it has been very reassuring to me to know that... if I do a little work I can be much better informed about my children's health."

For addressing more acute health conditions, the findings suggested the presence of two approaches to pediatric information seeking. One group, composed primarily of recent first-time mothers, reported that they regularly turned to the Internet for advice when their child had acute symptoms, when the doctor's office was not as reachable, and when making decisions about going to the emergency room. In contrast, the other group, composed primarily of older mothers, mothers of older children, and those with more than one child, reported that they were more likely to look in books or to call the doctor's answering service than to go online for health information. As one participant noted, "[Reading online pediatric information is] not like having a book. For me, I like having those books at the head of the bed. Because you have your little light on while your spouse is sleeping and you can look at stuff... I still find the computer to be like, yuck. It's not something you can cuddle up with."

Many participants reported turning to the Internet for advice on parenting issues and to seek social support from other mothers. This was particularly common among participants who worked at jobs outside of the home. Several of the working mothers noted that they used the Internet to solicit specific advice or share stories with other mothers, many times with women they only knew over the Internet. As one woman said, "It's nice to have all of that feedback from these other people so that you don't feel like you're alone."

In contrast, many stay-at-home mothers reported that they used the Internet to confirm their beliefs or to reassure themselves that their perceptions were correct. For example, several participants reported that they had looked to the Internet for reassurance that their child's development or behavior was "normal" and some participants sought support online for their ideas about parenting. In some cases, mothers were seeking support for their own beliefs to counter conflicting advice being offered by their pediatrician, and in other cases mothers were seeking a "second opinion" to confirm that advice received from their pediatrician was supported by others, especially other parents. See Table 1 for a list of the key reasons that participants gave for seeking online pediatric information.

**Table 1:** Reasons that mothers sought pediatric health information

To learn about diagnosing and treating pediatric conditions
To clarify or supplement messages received from practitioners or other sources
To confirm or provide reassurance for existing knowledge or beliefs
To seek advice or support on parenting issues

### Sources for pediatric health information

Participants reported that the most common way of finding pediatric health information on the Internet was through search engines, and when they found websites that they liked they would often return to the same sites. Other common ways in which the participants reported finding websites with health information were through word-of-mouth recommendations and through advertisements and articles in other media, especially parenting magazines.

When asked which websites they visit most frequently for health information, the most popular websites named by the participants fell into the category of *commercial information websites,* and included such examples as BabyCenter.com, ParentsPlace.com, and WebMD.com. Although these websites display commercial advertisements and often sell products, the participants did not see this as a potential conflict of interest. Said one participant: "The purpose of [one commercial information site] I think is to sell you stuff but they answer questions and it seems to me like that information sounds right." Many participants found these sites to be comprehensive and convenient and they liked the fact that they could shop, socialize, and research a wide range of topics all from the same website.

*Organizational websites* run by not-for-profit entities, and *academic websites*, run by universities and medical centers also were frequently mentioned as a good place for pediatric health information. Many participants praised these kinds of websites for containing reliable information, but some expressed displeasure that information on these sites, particularly the academic websites, can be too scientific and hard to understand. One mother complained about "...not being able to find what you're looking for and the frustration at ending up in 'journal level detail' when you're wanting 'mom level detail,' but [ideally] you want 'mom level detail' from an expert."

The *commercial product websites* maintained by companies selling specific baby products such as diapers, infant formula, or baby food were the least preferred type of health information website. Many participants reported that they had received direct mail or e-mail from such companies. However, few participants had, or would, go to these sites for health information, primarily because they were perceived to have an "ulterior motive" that undercuts the reliability of the health information they provide. This sentiment was more common among stay-at-home mothers than those mothers who worked outside the home. One mother who was critical of these websites representatively opined, "If [a major baby food manufacturer] sponsors [the website then] they just want to sell this stuff to me."

### Determining trust of pediatric health information

Trusting the reliability of information on the Internet was expressed as a serious concern by many focus group participants. When they were asked how they determine which websites to trust for health information, several strategies of determining trust emerged. One strategy involved trying to *determine the motives* of the website owners. If the primary purpose of an organization's website is perceived as the sale of their products, many mothers expressed skepticism for the trustworthiness of the health information presented there. In contrast, if the organization presenting the online information has little or nothing to gain financially by putting out the information, they are more likely to be trusted. In a similar vein, when asked about the relevance of different domain designations (i.e., .edu, .org, .net, .com) to trustworthiness, some participants noted that the Internet domain type can affect their perceptions of trust. As one participant observed, "A university setting I think...has more truth than a dot-com."

Another strategy that participants mentioned for determining the trust of a health website was to try to *identify and evaluate the source of the information* being presented. On many websites, the source of any given page or piece of information may be an individual writer, an organization, or even a different website, but some participants noted that discerning the original source of online information can be difficult. When the source could be determined, the most trusted sources, according to almost all the participants, were physicians and nurses, particularly when the information related to specific illnesses or conditions.

It was also noted that perceived trust in specific web-based sources of pediatric health information could increase over time as readers became more familiar with the source. For example, one participant who frequently visits a commercial information site, for example, described her perceptions of a pediatrician contributor to the site as follows: "I recognize him and his name because I read a lot of stuff that he writes [and] it is almost like another pediatrician to have available... I get to know him the same as you would [if you] have your own personal relationship." Along the same lines, some participants liked it when the source's name and picture were included. For example: "I think for me, psychologically, I like to see a person's face, see what they look like, just because it helps me to decide if I trust them or not."

In contrast, however, perceptions of source trustworthiness can break down when participants observe disagreements among the experts. For example, one mother noted, "...All of these pediatricians are basically contradicting themselves and they are experts. So if they're experts, why should we listen to them when they are contradicting each other? But that's one thing good about the Internet. You get on and you see all of these different ideas and you realize these guys aren't real experts. It makes you feel better as a mom."

The other highly trusted source for pediatric health information on the web was other parents, but only in specific situations. Other parents were commonly seen as a good source of support, reassurance, and advice on behavioral issues and parenting tips, but their advice was considered more suspect on issues of medical diagnoses and treatment. For example, when considering a webpage on pediatric skin cancer prevention written by a parent, one participant observed, "On this topic, I don't think what other parents have to say is of any use to me. On other things I think it is, but not on this subject."

Finally, the other strategy participants reported using to assess the trustworthiness of health information websites had to do with *information repetition and convergence*. Several participants reported that information appearing many times in many places is often considered to be more trustworthy than information that is not repeated. As one mother noted, "If you can find it in five or six spots [then] in my mind it is more likely that it is probably valid than if you read it one place but no one else is corroborating that or agreeing with it."

Information convergence with other non-Internet reference sources was also related to perceptions of trust. Almost all the participants reported that they would believe pediatric health information to be true if they received consistent information about it on the Internet, from their doctor, and from other parents. One participant described her process of seeking information convergence as follows: "I tend to use books first and talk to people second like [other] mothers... or relatives and then thirdly either go to the Web first to try to find more complimentary information and then check with the pediatrician." Another noted, "It doesn't matter what I read in a book or what I look up on the net, I'm going, in the end, if it is a health issue, [to] go ask my doctor. And if I don't like his answer, I'll ask another doctor." See Table 2 for a list of the strategies participants gave for determining the credibility of pediatric websites.

**Table 2:** How mothers determined trust of pediatric health information

Determine the motives of the website providers
Identify the source of the information being presented
Look for repetition and convergence of information from multiple sources

## Discussion

Understanding why, where, and how parents use the Internet to obtain pediatric health information is particularly important for caregivers and health educators because parents are turning to this information source with increasing frequency. Madden and Raine estimated that 80% of all Internet users, or more than 70 million Americans in December 2002, sought health information online [12]. About half of these online health information seekers are thought to have been seeking health information for themselves and the other half were searching for health information for someone else [13]. Almost 60% of parents with Internet access are active seekers of online health information [3]. These rates are likely to increase as more and more families obtain Internet access from home and more pediatric websites become available online.

The mothers in our study reported going online to learn more about the stages of fetal and child development, especially for their first child. This finding suggests that pre-natal and post-natal women can be high information seekers and that these time periods offer important opportunities for reaching mothers and mothers-to-be with essential pediatric health information. The information that these women found most useful were messages that were matched, or tailored, to their specific stage of pregnancy or the developmental stage of their child. The preferences these mothers expressed for personalized messages are consistent with other studies that have found strong preferences for tailored health messages over generic health messages [14-16]. Recent studies have found that web-based tailored messages are more effective than non-tailored web messages at changing participants' health-related beliefs and behaviors, [17,18] but there have been no known studies on the effectiveness of web-based tailored interventions for pregnant women or new mothers. This potential opportunity for effective prenatal and postpartum interventions warrants additional attention and research.

The primary reasons given by the mothers in our study for going online for health information were to research specific conditions or symptoms that their child was experiencing, and to get advice and support on parenting related issues. These online information seeking behaviors are consistent with studies of other Internet-using populations which have found that Internet users frequently seek online information on specific health conditions [19] and that they seek advice and social support for the different specific situations and challenges they face [20]. Our findings also revealed that the mothers' most trusted sources for these two types of information are different; not surprisingly, pediatricians and pediatric nurses were described as the most trusted source for online pediatric health information and other parents as the most trusted source for advice on parenting and for social support.

Our study participants expressed a great deal of overall skepticism and concern about the trustworthiness of sites, especially those that focus on selling specific baby-related products, which are similar to those found in other studies of Internet users that have revealed that many people do not trust or believe much of the information they receive online [21-22]. One study similar to this one that explored perceptions about online human genetics communication among Internet users found that most users would take the health information they received on the Internet with a "grain of salt." [23] 

Previous research with Internet users found multiple strategies for assessing the credibility of health related websites [7]. The most trusted sites included those from official authorities, and with professional layouts, understandable writing, and appropriate source citations [7]. An alternative strategy for assessing credibility that was revealed in our study was the use of repetition, i.e., many participants reported a greater trust of online health information when it was repeated on multiple websites or when the information was discovered to be consistent across multiple communication channels. This finding is consistent with research that has found that online health information seekers often feel reassured by advice they find that is repeated at more than one site and that matches what they already knew [16]. Using this type of "information convergence" strategy to establish accuracy of health information can be a useful way to confirm that ideas or opinions are widely held and accepted, especially when multiple sources of information are considered such as personal pediatricians, books, and peer-reviewed articles. This approach may be somewhat less effective, however, when convergence is sought only for unregulated information that appears on the web.

Due to the World Wide Web's vast size and unregulated nature, websites are likely to exist that advocate almost every conceivable position on any controversial pediatric topic. Therefore, if a parent seeks a supporting opinion for a potentially dangerous or inappropriate course of treatment for their child, they are likely to find multiple websites advocating that position if they look hard enough. To help prevent this from happening, practitioners should be aware that parents often seek "second opinions" online, especially when they don't like or understand what they have been told. Practitioners may consider recommending specific websites to their patients and their families that are known to be accurate, trustworthy, and consistent with best practices. Experts recommend that Internet health seekers allow ample time for conducting a thorough search, visit at least four different sites, determine the sponsor of each online health site, and identify the date when the information was last updated [19]. 

A number of limitations should be considered when interpreting the data presented in this study. As with most qualitative research, caution should be exercised in generalizing our findings to all mothers or other populations of mothers of young children. In addition, focus group research uses self-reporting techniques, and it is possible that some participants' comments are inconsistent with their actual experiences. Participants in this study had higher than average educational levels and were all from the greater metropolitan area of one medium-sized city in the Southeastern US. In addition, the sample had minimal racial and ethnic diversity, likely a result of the locations from which the participants were recruited. All participants were required to have used the Internet at least one time in the previous 6 months, but they were not required to have specifically looked up pediatric health information during that time period. Although almost all participants acknowledged viewing pediatric information online, it is possible that some participants' comments referred to older or more theoretical experiences. In addition, only mothers were recruited for this study because women are twice as likely as men to seek online health information for their children, [2] and it is possible that fathers have considerably different experiences and perceptions about online health information. Finally, the number of focus groups conducted and the overall number of participants was relatively small. Despite the fact that the data demonstrated considerable convergence, it is entirely possible that other important opinions and perspectives were missed.

In conclusion, the findings from this study suggest that the pre-natal and post-natal periods may represent "teachable moments" when women are high information seekers for online pediatric information. Because there are countless pediatric health-related websites available presenting widely divergent opinions of varying degrees of quality and accuracy, it is important that practitioners be educated about high-quality, accurate pediatric health websites and encourage their patients to avoid inappropriate websites. Participants largely preferred websites with tailored health information, pediatric content that is presented by caregivers, and parenting advice that is presented by other parents. Information that is repeated consistently across multiple sources or websites may increase perceptions of trust. Future research should seek to replicate and expand upon these findings with more diverse populations and through quantitative surveys administered to larger and more generalizabile samples of mothers and fathers.

## Multimedia Appendix

Focus Group Guide:
