# Supplementary material for: Online Pediatric Information Seeking Among Mothers of Young Children: Results From a Qualitative Study Using Focus Groups
Source: J Med Internet Res. 2004 Mar 1;6(1):e7. doi: 10.2196/jmir.6.1.e7 (PMC1550581; doi:10.2196/jmir.6.1.e7)
Supplement: Supplementary file 1 [file jmir_v6i1e7_app1.pdf]

Online pediatric information seeking among mothers of young children:
Results from a qualitative study using focus groups

Focus group guide

NOTE: Probes were used only when topic did not come up in general discussion or to prolong or adjust the length of a particular discussion.

1. Introduction and Icebreaker
2. What do you think of when I say the word "Internet?"
   - 2.1. How did you find out about the Internet?
   - 2.2. What were your early impressions?
3. Have you ever used the Internet to look up health information for your kids?
   - 3.1. What parts of the Internet did you use to look up health information? (ie, the Web, email, chatroom, bulletin board)
   - 3.2. Can you give an example?
   - 3.3. Have you ever used any other parts of the Internet to look up health information for your kids? When? Where? Why? For what?
4. We've been talking about a lot of different ways that people use the Internet, like email, chatrooms and bulletin boards. I'd like to focus on the Web for the rest of our conversation.
   - 4.1. Why did you look for health information for your kids on the Web?
     - 4.1.1. What were you looking for?
     - 4.1.2. Have you ever looked up information when they were sick? What about when they were healthy? When? Why?
     - 4.1.3. Have you ever looked up medical information "after hours" when you couldn't talk to the doctor?
     - 4.1.4. Do you think you will use it more or less as your children get older? Why?
5. How do you decide what site to go to on the Web?
   - 5.1. What are some ways that you have found information on the Web?
   - 5.2. Have you ever gone to a site that you've seen linked off another site? Can you give me an example?
   - 5.3. What sites have you heard about from another person? Who? What was the site?
   - 5.4. What search engines do you use?
   - 5.5. What sites have you seen advertised? What is the site for? Where was it advertised?
6. Is the Web a good source for health information? Why? Why not?
   - 6.1. How much of the information you see on the Web is true?
   - 6.2. Have you had a specific experience where you have found untrue information on the Internet?
   - 6.3. How do you decide if a particular site has truthful information?
   - 6.4. Does the domain (the .com or .gov or .edu or .org ending on the address) make a difference to you?
   - 6.5. Do you think information you look up on the Web is private? Confidential?
   - 6.6. How important is it to you that data on the Web be private? Confidential?
7. Now let's talk about what specific sites you like to get health information on the Web from, based on your experience. Do any of you have favorite sites?
   - 7.1. What about that site do you like?
   - 7.2. Who writes the information on the site?
   - 7.3. Does it matter who writes the articles?
   - 7.4. Would you rather read articles by a doctor, nurse, professor, parent? Why
   - 7.5. Have you ever been on a site that you really believed the information? Which one? What made you believe in it?
   - 7.6. Have you ever been on a site that you really trusted? Which one? What made you trust it?
   - 7.7. Have you ever been on a site that you really liked? Which one? What made you trust it?

Focus group guide (continued)

Now let's talk about what kinds of health information for your kids you look for.

8. What are topics that you have looked up?
   8.1. Are there some topics that are easier to find than others on the Web?
   8.2. What are some of the good things that you have found on the Web? Why?
   8.3. What are some of the good things about using the Web to look for health information?
   8.4. What are some of the bad things about using the Web to look for health information?
9. We've been talking a lot about all kinds of different health topics on the Web. Now we're going to talk about sun protection for kids, just to give us an example of a health topic.
   9.1. First of all, what comes to mind when I say "sun protection for kids?"
   9.2. Have any of you looked up information on sun protection on the Web before? Where? Why? How?
10. Now I'm going to show you some examples of Web pages. You may or may not have seen these pages before. I'm going to project them up on the screen, and then let's talk about what you think of them.
    10.1. Ivillage.com (http://www.parentsplace.com/health/babycare/qa/0,3435,1039,00.html)
    10.2. Center for Disease Control's skin cancer page (http://www.cdc.gov/mmwr/preview/mmwrhtml/00053602.htm)
    10.3. American Cancer Society (http://www.cdc.gov/mmwr/preview/mmwrhtml/00053602.htm)
    10.4. What is the first thing you notice on the page?
    10.5. Who wrote the information on this page? Is it clear?
    10.6. What do you like about this page?
    10.7. What don't you like about this page?
    10.8. How much time would you spend on this page?
    10.9. Would you like more or less text? More or less graphics?
    10.10. Does this page seem interactive to you? Would you like it to be more or less interactive?
    10.11. Does this page seem personalized to you? Would you like it to be more or less personalized?
